# Harnessing Vδ1^+^ T cells for next-generation immunotherapy: from mechanistic insights to clinical translation

**DOI:** 10.3389/fimmu.2026.1827307

**Published:** 2026-06-09

**Authors:** Fan Wang, Jiaxuan Zhao, Tongcun Zhang, Jiangzhou Shi

**Affiliations:** 1 Institute of Biology and Medicine, College of Life Science and Health, Wuhan University of Science and Technology, Wuhan, Hubei, China; 2 College of Biotechnology, Tianjin University of Science and Technology, Tianjin, China; 3 School of Metallurgy and Energy, Wuhan University of Science and Technology, Wuhan, China

**Keywords:** Vδ1^+^ T cells, tumor immunotherapy, MHC-unrestricted antigen recognition, adoptive cell therapy, tumor microenvironment

## Abstract

The unique ability of Vδ1^+^ T cells to recognize targets in a major histocompatibility complex (MHC)-unrestricted manner makes them promising candidates for immunotherapy. This review explores the biological underpinnings of their advantages, particularly their ability to penetrate and migrate through solid tumors, their antigenic promiscuity, and their functional plasticity in inflammatory tumor microenvironments. Based on this, we analyze the therapeutic pipeline derived from this biology, including targeted antibodies, bispecific molecules and a new generation of cellular products, among which chimeric antigen receptor (CAR)-engineered constructs have shown encouraging clinical responses. Nonetheless, translation into the clinic can often present challenges; we tackle the unresolved issues of product standardization, subset complexity, and microenvironmental suppression, offering practical ways forward. This review aims to serve as a reference to deepen the understanding of the anti-tumor value of Vδ1^+^ T cells and facilitate their translational development.

## 1 Introduction

Tumor immunotherapy faces two key problems that limit its effectiveness in the clinic (1). First, the high molecular homology between tumor cells and normal somatic cells hampers the immune system’s ability to distinguish “self” from “non-self” thus disrupting specific recognition and efficient elimination of malignant cells (2, 3). Second, the tumor microenvironment (TME) impairs immune function through various mechanisms and induces immune escape (2–4). Although immune checkpoint blockade (ICB) and chimeric antigen receptor T cell (CAR-T) therapy have brought about a revolution in the treatment for certain malignancies (5, 6), there are still a large number of patients and tumor types lacking effective immunotherapeutic options (7). Downregulation of major histocompatibility complex (MHC) class I molecules is a major immune escape mechanism in advanced tumors. This event compromises MHC-dependent immunotherapies using αβ T cells and acts as a critical bottleneck for conventional treatments (8). γδ T cells, however, circumvent this limitation through their mode of recognition that is mediated by their T cell receptor (TCR) and independent of MHC expression (9). γδ T cells make up a very small percentage of circulating lymphocytes. However, they have a natural tendency to migrate towards tumor sites, especially epithelial-derived tumors. Their inhibitory effects on tumor proliferation and metastasis have been reported (9). Therefore, understanding the crosstalk between tumors and γδ T cells, particularly in terms of MHC deficiency, is important for developing next-generation immunotherapies. This line of research contributes to the basic understanding of tumor-immune interactions, which provides a theoretical basis for clinical translation, underscoring that dissecting γδ T cells in tumor evolution has important clinical value.

γδ T cells are a unique lymphocyte subpopulation capable of linking innate and adaptive immunity (10–13). Compared to conventional αβ T cells, they offer distinct advantages for cancer immunotherapy, primarily stemming from their MHC-independent antigen recognition. This property allows tumor targeting through alternative mechanisms (14, 15). It markedly lowers the risk of graft-versus-host disease (GVHD) by avoiding MHC-restricted activation (16). Furthermore, genetically engineered γδ T cells exhibit potent anti-tumor efficacy comparable to αβ T cells while producing substantially lower levels of cytokines, which is expected to reduce the clinical incidence of cytokine release syndrome (CRS) (17–19). In addition, as early crucial producers of IFN-γ, γδ T cells amplify their own anti-tumor responses and modulate adaptive immunity by altering αβ T cells, dendritic cells and B cells, leading to an overall anti-tumor immune response of αβ T cells (20).

In contrast to natural killer (NK) cells, γδ T cells exhibit superior attributes in anti-tumor immunotherapy. γδ T cells possess a stronger capacity for infiltrating the tumor microenvironment and exhibit relatively weaker regulation of killer inhibitory receptor expression, which are pivotal factors that restrain NK cell activation. This distinctive molecular expression pattern is considered crucial for their anti-tumor efficacy (21). Moreover, regarding their functional mechanism, γδ T cells integrate the TCR-mediated immune response characteristic of αβ T cells with the receptor signaling pathways of NK cells. This dual mechanism enhances their tumor targeting specificity and may improve the therapeutic outcome (7).

Collectively, γδ T cells are MHC-unrestricted and adaptable to the tumor microenvironment, and thus present a promising strategy to overcome the hurdles of classical cancer immunotherapy. However, various challenges persist, ranging from functional heterogeneity due to subtype diversity to antigen recognition mechanism complexity and technical barriers to clinical translation. Importantly, growing evidence suggests that the Vδ1^+^ T cell subset displays distinct biological and translational advantages, particularly in solid tumor settings, attributable to its tissue residency, enhanced intratumoral infiltration, and sustained functionality within immunosuppressive microenvironments. Accordingly, this review centers on Vδ1^+^ T cells and integrates recent advances in antigen recognition, adaptation to the tumor microenvironment, and translational application in cancer immunotherapy. We emphasize how the biological characteristics of Vδ1^+^ T cells contribute to their therapeutic potential. By linking these features to emerging therapeutic strategies and the challenges encountered during clinical translation, this review aims to provide a structured resource that supports both mechanistic understanding and the rational development of Vδ1^+^ T cell–based immunotherapies.

## 2 Classification and antigen recognition of γδ T cells

### 2.1 Classification of γδ T cells

Human γδ T cells can be classified into four main types based on the composition of their TCR δ chain, i.e.: Vδ1^+^, Vδ2^+^, Vδ3^+^ and Vδ5^+^ (22–24) (**Table 1**). Among them, the Vδ1^+^ and Vδ2^+^ subsets dominate the γδ T cell population (25–28), and each subtype differs in tissue distribution and physiological functions.

**Table 1:** Characteristics of human γδ T cell subsets.

δ Chain	γ Chain	Distribution	Anti-tumor effector molecules	Antigen recognition
Vδ1	Vγ2, Vγ3, Vγ4, Vγ5, Vγ8, Vγ9, Vγ10	Spleen, liver, dermis, intestines, and other epithelial tissues, peripheral blood	Perforin, granzyme, IFN-γ, TNF-α, FASL, TRAIL	(1) CD1d-dependent recognition of lipid antigens. (2) NKR-mediated: NKG2D (binds MICA/MICB/ULBPs). (3) TCR-independent/antigen-free: binds annexin A2/A6, EphA2, MR1; Vγ4 chain associates with BTNL3/BTNL8.
Vδ2	Vγ9	Peripheral blood	Perforin, granzyme, IFN-γ, TNF-α, FASL, TRAIL	(1) BTN-dependent recognition of prenyl pyrophosphate metabolites *via* TCR. (2) NKR-mediated: NKG2D/DNAM1 (binds MICA/MICB, ULBP-2, nectin-2, PVR).
Vδ3	Vγ2, Vγ3	Liver, intestines, peripheral blood	Unknown	(1) CD1d-restricted: Interacts with CD1d (with/without antigens) through TCR. (2) Antigen-loading independent: Recognizes annexin A2 or MR1.
Vδ5	Vγ4	Peripheral blood	Unknown	(1) TCR-mediated: Specifically binds to EPCR (endothelial protein C receptor).

This table summarizes the tissue distribution, anti-tumor effector molecules, and antigen recognition mechanisms of major human γδ T cell subsets (Vδ1^+^, Vδ2^+^, Vδ3^+^, Vδ5^+^).

FASL, human apoptosis-related factor ligand; TRAIL, tumor necrosis factor-related apoptosis-inducing ligand; NKRs, natural killer cell receptors; NCRs, natural cytotoxicity receptors; MR1, MHC-related protein 1; BTN, butyrophilin; TCRs, T cell receptors; PVR, polyoma virus receptor; EPCR, endothelial protein C receptor.

Vδ1^+^ T cells are mainly found in the mucosal tissues (intestinal tissues) and epithelial tissues (such as skin), although smaller cell populations can also be detected in the liver and spleen (29, 30), accounting for approximately 15% of γδ T cells in peripheral blood mononuclear cells (PBMCs) (31). The Vδ1 chain can pair with multiple γ chains (*e.g.*, Vγ2, Vγ3, Vγ4), forming heterogeneous subsets that display distinct functional properties (32). The apoptosis of tumor cells mediated by these cells occurs through the activity of cytotoxic mediators such as perforin, granzyme, and granulysin, as well as key cytokines including IFN-γ and TNF-α (33). Numerous studies have shown strong cytolytic anti-tumor activity mediated by these mechanisms in cancerous cells. These malignancies include B-cell chronic lymphocytic leukemia (B-CLL) and acute myeloid leukemia (AML) in hematological cancers, melanoma and neuroblastoma in solid tumors (34–37).

The most abundant T cell subtype in human peripheral blood is the Vδ2^+^ T cell. It accounts for approximately 50% to 95% of total γδ T cells (38). The Vδ2 chain during TCR γδ gene rearrangement shows very high pairing specificity. Most predominantly, it pairs with the Vγ9 chain forming a functional heterodimer, Therefore, this cell is also known as Vγ9Vδ2 T cell (39). They are capable of hindering the cycle of tumor growth and inducing tumor cell apoptosis, thus contributing greatly to tumor suppression (40).

Vδ3^+^ and Vδ5^+^ T cells are relatively rare subsets of γδ T cells that have not been thoroughly studied. Vδ3^+^ T cells constitute a small fraction of peripheral blood lymphocytes in healthy individuals but are strongly enriched in liver and intestinal mucosal epithelium (41–45), and mainly pair with Vγ2 or Vγ3 chains (46). Vδ5^+^ T cells are a very uncommon cell type. The research group led by Willcox was first to identify the Vγ4Vδ5 T cell clone (26). Although Vδ5^+^ T cells can be found in peripheral blood and tissues, their precise physiology has not yet been fully defined (26, 46–49).

### 2.2 Antigen recognition of γδ T cells

#### 2.2.1 Antigen recognition of Vδ1^+^ T cells

**Figure 1:**
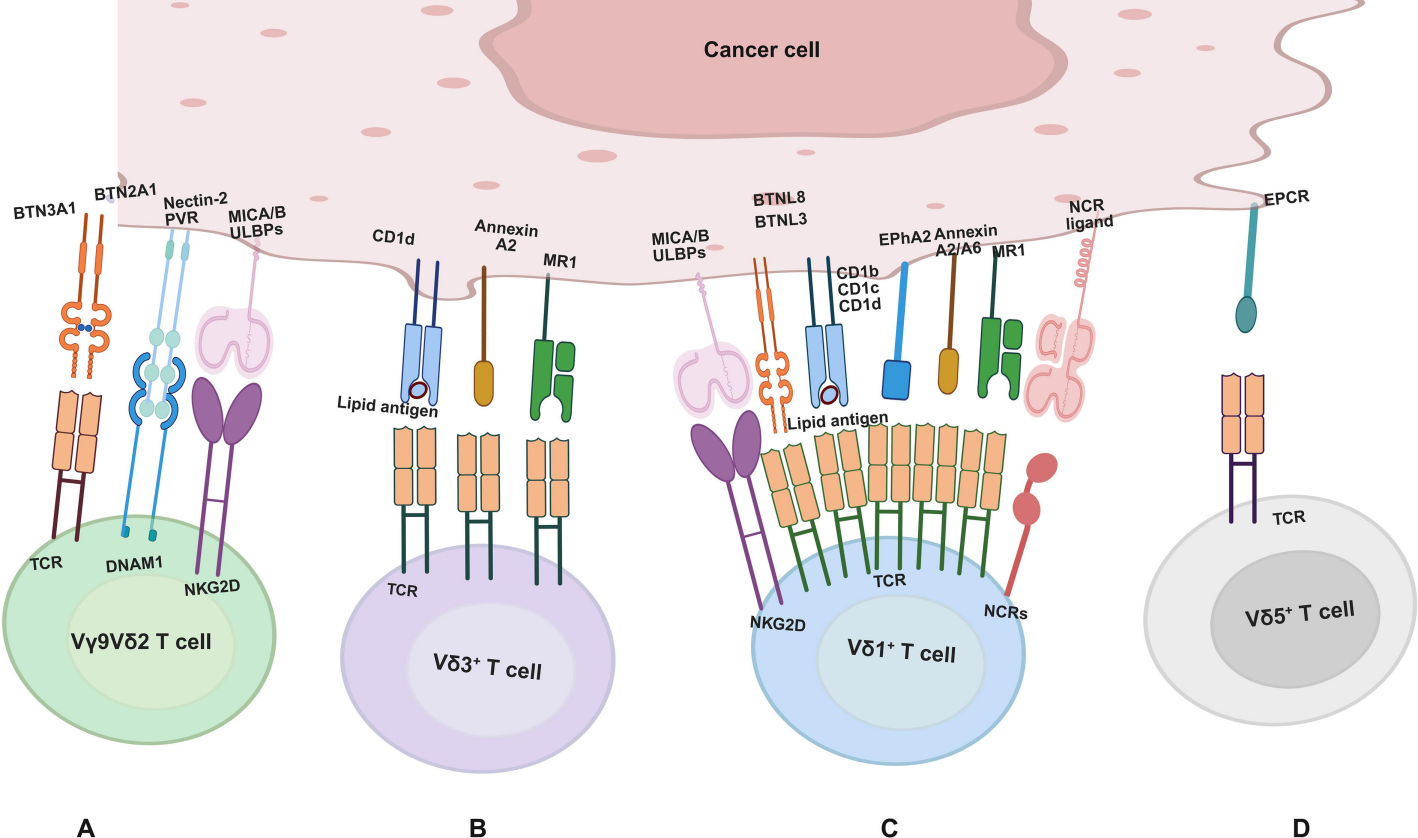
Ligand recognition landscape of human γδ T cell subsets. **(A)** Vγ9Vδ2 T cells: TCR-mediated recognition of prenyl pyrophosphate metabolites in a BTN-dependent manner. Stimulation is *via* NKG2D and DNAM-1 receptors binding stress ligands (MICA/B, ULBPs) and adhesion molecules (Nectin-2, PVR), respectively. **(B)** Vδ3^+^ T cells: TCR-dependent engagement of CD1d, and antigen-independent interaction with annexin A2 or MR1. **(C)** Vδ1^+^ T cells: Diverse mechanisms include TCR-mediated recognition of CD1-presented lipids; NKG2D-dependent sensing of stress ligands (MICA/B, ULBPs); and antigen-independent interactions with annexin A2/A6, EphA2, MR1. A unique Vγ4-mediated pathway engages BTNL3/BTNL8. **(D)** Vδ5^+^ T cells: Direct, TCR-mediated recognition of EPCR. BTN, butyrophilin; BTNL, butyrophilin-like protein; CD1d, cluster of differentiation 1d; DNAM1, DNAX accessory molecule 1; EphA2, ephrin type-A receptor 2; EPCR, endothelial protein C receptor; MICA/B, MHC class I chain-related protein A/B; MR1, MHC class I-related protein 1; NKG2D, natural killer group 2, member D; Nectin-2, Nectin cell adhesion molecule 2; PVR, poliovirus receptor; TCR, T cell receptor; ULBPs, UL16-binding proteins.

Vδ1^+^ T cells produce a variety of immune responses through multiple antigen recognition mechanisms (**Table 1**; **Figure 1**). The ability to engage CD1 family molecules (CD1b, CD1c, CD1d) in a lipid antigen-independent manner is critical for their function (50–54). Moreover, they make use of natural killer receptors (NKRs), like NKG2D that recognizes stress ligands MICA, MICB and ULBPs thereby mediating lysis of tumor cells (55–58). Apart from these, detection of tumor cells also occurs through natural cytotoxicity receptors (NKp46, NKp44, NKp30) expression. However, this expression is context dependent and can be enhanced using cytokines such as IL-15 or IL-2 (29, 59). A significant autoreactivity towards MHC-related protein 1 (MR1) indicates that this molecule may serve as an endogenous ligand (60). At the level of the TCR, the Vγ9Vδ1 receptor recognizes EphA2 for tumor killing, a mechanism underscored by the significant reduction in cytotoxicity upon EphA2 blockade (61, 62). Moreover, annexin A2 and A6 induce proliferation of the Vγ4Vδ1 subset and cause it to secrete various cytokines like TNF-α (49). Furthermore, the binding of Vγ4 to BTNL8 and BTNL3, two subtypes of the butyrophilin (BTN) family of molecules, specifically induces immune activation (63).

#### 2.2.2 Antigen recognition of Vγ9Vδ2 T cells

The antigen recognition mechanisms of Vγ9Vδ2 T cells are broadly categorized into TCR-mediated and NKR-mediated recognition (9) (**Table 1**; **Figure 1**). In the TCR-dependent pathway, these cells respond to the intracellular accumulation of prenyl pyrophosphate metabolites, such as isopentenyl pyrophosphate (IPP), which is a small-molecule intermediate of the mevalonate pathway (41). Notably, Vγ9Vδ2 T cells do not directly recognize these metabolites; instead, sensing is mediated by butyrophilin (BTN) family molecules. BTN3A1 functions as an intracellular sensor of prenyl pyrophosphates via its B30.2 domain, where ligand binding induces conformational changes that are transmitted to the extracellular domain. These changes are thought to reorganize the BTN3A1–BTN2A1 complex, enabling BTN2A1 to facilitate Vγ9Vδ2 TCR engagement and activation (64–67). In addition, Vγ9Vδ2 T cells express natural killer cell receptors, such as NKG2D and DNAM-1, similar to their Vδ1^+^ counterparts. Stress-induced ligands, MICA/MICB and ULBPs, which are targets of the NKG2D receptor may enable target cell recognition (68, 69). In contrast, the DNAM-1 receptor binds to ligands, including nectin-2 and polyoma virus receptor (PVR) molecules that are often overexpressed on tumors (68, 70).

#### 2.2.3 Antigen recognition of other γδ T cell subsets

Relative to Vδ1^+^ and Vγ9Vδ2 T cells, there are relatively few studies on the antigen recognition methods of other subsets. Vδ3^+^ T cells can specifically recognize CD1d molecules but cannot recognize CD1a, CD1b, and CD1c, and exhibit cytotoxicity against CD1d-positive targets when activated (71). This subset also displays an antigen-independent capacity to recognize MR1 molecules (45). Furthermore, Vγ8Vδ3 expands this recognition repertoire by directly binding to annexin A2 (49). With respect to the limited Vδ5^+^ subset, it was demonstrated that its TCR directly engages the endothelial protein C receptor (EPCR), an MHC I/CD1-like molecule (26, 72) (**Table 1**; **Figure 1**).

The different pathways for the recognition of antigens by γδ T cell subsets play a fundamental role in their anti-tumor potentials. While Vγ9Vδ2 T cells primarily respond to prenyl phosphates and Vδ3^+^ T cells largely rely on CD1d recognition, Vδ1^+^ T cells exhibit the broadest mechanistic repertoire. Vδ1^+^ T cells eliminate tumor cells and modulate the immune response through multiple mechanisms that includes recognition through CD1, engagement of the NKG2D receptor, and binding to MR1, BTNL8/BTNL3, and EphA2. Among these subsets, the exceptional diversity of antigen recognition and immunomodulatory mechanisms uniquely positions Vδ1^+^ T cells as a particularly versatile platform for cancer immunotherapy.

## 3 Advantages of Vδ1^+^ T cells in tumor immunotherapy

With the development of tumor immunotherapy at an accelerated pace, the functions of γδ T cells have received growing interest, with immunoregulatory functions of Vδ1^+^ and Vγ9Vδ2 T cells being the focus of research. Early γδ T cell immunotherapy investigations have been preferentially studied with Vγ9Vδ2 T cells because of their diverse tumor recognition receptors and robust proliferation ability *in vitro (*73–75). Nevertheless, cumulatively with the deepening understandings of the TME and immune response mechanisms, the biological characteristics and functional significance of Vδ1^+^ T cells have been progressively clarified. Although they account for a relatively small fraction of γδ T cells (76), mounting evidence validates their critical role in anti-tumor immunity (77, 78). In comparison to Vγ9Vδ2 T cells, Vδ1^+^ T cells not only have a broader antigen recognition spectrum and greater advantages related to tissue residency and mucosal immunity but also exhibit superior biological characteristics in key dimensions closely related to tumor immunotherapy, such as infiltration ability, TME adaptability, functional persistence and exhaustion resistance. These characteristics of Vδ1^+^ T cells make them especially attractive to study in a tumor immunotherapy setting.

### 3.1 Multiple anti-tumor mechanisms and broad-spectrum targeting

In contrast to Vγ9Vδ2 T cells, Vδ1^+^ T cells exhibit distinct anti-tumor advantages attributable to their specific antigen recognition mechanisms and functional profile. Notably, they possess multifaceted effector strategies and the ability to target a broader spectrum of tumors. First, Vδ1^+^ T cells enhance anti-tumor responses through the expression of natural cytotoxicity receptors (*e.g.*, NKp30, NKp46) (29, 57). In a human colon cancer xenograft mouse model, the anti-tumor activity of Vδ1^+^ T cells was significantly greater than that of Vγ9Vδ2 T cells, and this difference was confirmed to be directly related to the high expression of such receptors in the former (79). Second, Vδ1^+^ T cells exert potent antibody-independent cytotoxicity. Their killing capacity is equal to or greater than that of Vγ9Vδ2 T cells, even in the absence of opsonizing antibodies. In contrast, the cytotoxicity of Vγ9Vδ2 T cells is significantly reduced in the absence of opsonizing antibodies (80). Through various recognition pathways, including potential ligand interactions (81, 82), Vδ1^+^ T cells show broad targeting, particularly against MHC I-negative tumors. For example, tumors deficient in DNA mismatch repair (MMR-d) often lack MHC I expression, making them immune to conventional antigen-presentation-dependent immunity (83, 84). Vδ1^+^ T cells overcome this limit through receptors such as NKp46, NKG2C and NKG2D, allowing them to efficiently recognize and kill MHC I-negative targets, In contrast, reactivity of Vγ9Vδ2 T cells in this context is reportedly limited (14). Although various γδ T cells generally express effector molecules (granzyme B, perforin) (14), accumulating evidence consistently suggests that Vδ1^+^ T cells are the more cytotoxic subset (29, 79, 85, 86). Therefore, these advantages in recognition mechanisms and functional adaptability may support a central role for Vδ1^+^ T cells in tumor immunotherapy.

### 3.2 Advantages in tissue distribution and tumor infiltration

#### 3.2.1 Tissue residency and mucosal immunity

Although Vδ1^+^ T cells are uncommon in peripheral circulation, they are the primary tissue-resident γδ T cell subset (59) and are present in large numbers at mucosal sites (43, 87). This unique tissue distribution shapes their immune functions. According to a tumor immunology viewpoint, tissue-resident Vδ1^+^ T cells have been shown to directly respond to shared antigens present on epithelial-derived tumor cells. In response to this antigen recognition, these cells can mediate specific cytotoxicity against autologous and allogeneic tumor cells (85, 88), highlighting their functional value in immune surveillance of mucosal-derived tumors. The effector functions of Vδ1^+^ T cells in mucosal settings are closely associated with their activity in the defense against infection. The intestinal mucosa serves as the primary entry site during early HIV infection. In this context, Vδ1^+^ T cells display significantly higher CD107a expression compared to Vγ9Vδ2 T cells, indicating an enhanced degranulation capacity and suggesting a more pronounced cytotoxic effector function (89). Similarly, Vδ1^+^ T cells undergo specific expansion, when infected with cytomegalovirus (CMV) (90–92). The anti-CMV reactive Vδ1^+^ T cells can cross-recognize intestinal tumor epithelial cells and kill *via* both cytokine and perforin (93). Additionally, the larger Vδ1^+^ T cell population appears to drive stronger tumor immunity and a lower risk of tumorigenesis in kidney transplant recipients (94). Thus, this evidence further supports the functional connection between their anti-infection and anti-tumor defense in mucosal settings.

#### 3.2.2 Dual advantages of tumor infiltration and solid tumor breakthrough

Due to their association with epithelial tissues, Vδ1^+^ T cells show a greater tendency to express the receptor molecules associated with tissue homing and retention (14, 95, 96). This may explain their ability to infiltrate tumors and cross microenvironmental barriers. Indeed, flow cytometric assessment of tumor-infiltrating lymphocytes (TILs) shows a selective accumulation of Vδ1^+^ T in tumor tissue of esophageal cancer, in which surface adhesion molecules are critical for recruitment and retention (97). Further understanding of the mechanisms regulating their migration remains an important area of investigation. Together, this intrinsic tissue preference and directional migration capacity allow Vδ1^+^ T cells to effectively infiltrate tumors while overcoming the physical hurdles imposed by the tumor microenvironment and eliciting a strong anti-tumor response.

##### 3.2.2.1 Precise migration regulation endows Vδ1^+^ T cells with efficient tumor infiltration ability

Vδ1^+^ T cells have superior tumor infiltration capacity due largely to their effective migratory behavior, which is mediated by specific homing receptors and factors. When compared with Vγ9Vδ2 T cells, Vδ1^+^ T cells have an enhanced potential for tissue infiltration owing to their varied chemokine receptor profile (98). Mechanistically, Wu et al. reported that Vδ1^+^ T cells express a variety of chemokine receptors (including CCR4, CCR6, CCR7, CXCR1, CXCR5, and CXCR7), enabling a targeted ingress through the interaction with the chemokines present in the tumor microenvironment (79). In a model of transplantable B16 melanoma, CCL2 upregulation and specific activation of CCR2 by CCL2 in the TME led to Vδ1^+^ T cells migrating to the tumor site and producing a high level of IFN-γ (99). Furthermore, in breast cancer, IP-10/CXCR3 axis mediates the migration of Vδ1^+^ T cells (100), and CCR4/CCR8 interaction through the ligands CCL17/CCL22 which is thought to enable their recruitment to TME (101). The infiltration of Vδ1^+^ T cells into the tumor tissues is coordinated *via* multiple pathways.

##### 3.2.2.2 Breaking through solid tumor barriers enables potent anti-tumor effects of Vδ1^+^ T cells

In addition to efficient infiltration, Vδ1^+^ T cells display unique attributes that allow them to overcome the physical and immune barriers present in solid tumors. Unlike Vγ9Vδ2 T cells, Vδ1^+^ T cells can cross the fibrotic matrix and abnormal vasculature to directly reach the tumor core (41, 102–104). This deep infiltration ability is likely to lay a structural basis for their powerful anti-tumor functions.

In terms of functional activity, tumor-infiltrating lymphocyte (TIL) cultures obtained from Vδ1^+^ T cells were more cytotoxic than those obtained from Vγ9Vδ2 T cells (105, 106). This superior cytotoxicity is corroborated by early studies showing that Vδ1^+^ T cells isolated from various solid tumors and expanded *in vitro* display greater killing efficacy against both tumor cell lines and freshly isolated tumor cells compared to their Vγ9Vδ2 counterparts (85, 88, 107–110).

From the perspective of distribution within tumor tissues, Vδ1^+^ T cells are the most common γδ T cells in various solid tumors. For example, they comprise the most abundant TIL subset in colorectal cancer (CRC) and primary malignant melanoma (PMM), and contribute directly to anti-tumor immunity (111, 112). Interestingly, in CRC patients with liver metastasis, the largest subset of TILs is Vδ1^+^ T cells. The presence of these T cells corresponds with anti-tumor activity and a higher overall survival (113). Collectively, these clinical and functional observations indicate that Vδ1^+^ T cells play an essential role in tumor suppression after barrier penetration.

To sum up, effective migration regulation plus an intrinsic ability to breach barriers of solid tumors allows Vδ1^+^ T cells for efficient intratumoral accumulation and potent anti-tumor effector functions. This dual infiltration-breakthrough strength shows significant potential in immunotherapy against solid tumors. In particular, with respect to adoptive cell therapy, their properties offer great promise for addressing the key challenge of limited tumor bed penetration (114).

### 3.3 Functional plasticity advantages in the tumor microenvironment

The plasticity of Vδ1^+^ T cells in the TME is an important therapeutic asset for overcoming the main barriers to durable anti-tumor immunity. This adaptive ability is mainly exerted through three mechanisms: reversible immune checkpoint modulation, intrinsic resistance to exhaustion and adaptation to hypoxic niches.

#### 3.3.1 Reversible functional modulation by immune checkpoints

A unique aspect of Vδ1^+^ T cell regulation is reversible functional modulation that is mediated by immune checkpoints, notably PD-1. While these cells express programmed cell death protein 1 (PD-1), an established marker of T cell exhaustion (95, 96, 115, 116), this does not indicate irreversible functional deficiency (117). The findings of Davies et al. further revealed the particularity of Vδ1^+^ T cells: although PD-1^+^Vδ1^+^T cells and PD-1^+^CD8^+^αβT cells share some similarities in the exhaustion mechanism, they show key differences. Notably, PD-1^+^Vδ1^+^ cells can maintain the effector response ability during TCR signal activation, and this effector function is only temporarily inhibited by the PD-1 molecule, and the inhibitory effect of PD-1 can be effectively blocked by checkpoint inhibitors (CPI) (118).This mechanistic insight shows direct evidence that their ‘functional suspension’ state can be reversed and reactivated. In addition, significance of this reversibility is highlighted by definitive responses of Vδ1^+^ TILs to PD-1 inhibitor therapy in microsatellite instability colorectal cancer (MSI CRC), renal cell carcinoma (RCC), and Merkel cell carcinoma (MCC) (14, 75, 119). Also, this principle applies to other checkpoints: in microsatellite-stable colorectal cancer (MSS CRC), blocking the T cell immunoreceptor with Ig and ITIM domains (TIGIT)–Nectin cell adhesion molecule 2 (NECTIN2) axis with an anti-TIGIT antibody partially restores the cytotoxicity of dysfunctional Vδ1^+^ T cells (120).This characteristic provides a strong rationale for combining Vδ1^+^ T cells with immune checkpoint inhibitors (ICIs) for cancer immunotherapy to reactivate their anti-tumor effector functions.

#### 3.3.2 Exhaustion resistance and sustained effector function

A critical difference between the roles of Vδ1^+^ and Vγ9Vδ2 T cells in long-term anti-tumor activities is their fates upon activation: resistance versus susceptibility to functional exhaustion and activation-induced cell death (AICD). This differential AICD susceptibility is likely to be a key determinant (81, 121).

Vδ1^+^ T cells have a resilient phenotype and are significantly less susceptible to AICD than Vγ9Vδ2 T cells (121) and they do not show susceptibility to AICD in multiple experiments (122–124). In most *in vitro* assays and preclinical models, they exhibit increased cytotoxic activity and prolonged survival (59, 125). At the same time, Vδ1^+^ T cells can also provide additional support for the long-term maintenance of tumor immunity through long-term immune surveillance (58, 79, 81).

Vγ9Vδ2 T cells, in contrast, undergo a rapid loss of function. This subset is easily induced by AICD (126), which represents a significant limitation of therapies based on prolonged stimulation, such as those using aminobisphosphonates (127). Moreover, Vγ9Vδ2 T cells are relatively more differentiated and express markers of exhaustion (37). For patients, repeated stimulation causes functional decay, energy exhaustion and terminal differentiation leading to loss of anti-tumor ability (122, 128, 129).

The above differences clearly show that Vδ1^+^ T cells achieve long-term survival and function maintenance by virtue of anti-AICD characteristics, showing significant exhaustion resistance and functional durability. In contrast, Vγ9Vδ2 T cells have limited functional durability due to their high susceptibility to AICD and propensity for exhaustion characteristics, which highlights the important application potential of Vδ1^+^ T cells in long-term tumor immunotherapy.

#### 3.3.3 Adaptation to hypoxic TME

The TME is in a hypoxic state for a long time due to the rapid proliferation of tumor cells, enhanced metabolic activity, and relatively insufficient oxygen supply (130). This special environment that regulates the function of anti-tumor immune cells through signals such as oxygen tension and nutrient availability has a significant diversity of effects on the activity of γδ T cells (9). Specifically, in *in vitro* experiments, the hypoxic environment may promote the activity of γδ T cells, may show an inhibitory effect, or may have no significant effect (9, 131–134).

Vδ1^+^ T cells residing in tissues have the characteristics of adapting to low nutrient supply and low oxygen levels, and this adaptability is consistent with the characteristics of the tumor microenvironment (33). In the hypoxic tumor microenvironment, the Vδ1^+^ subset shows stronger tissue tropism and invasion ability and can more effectively infiltrate the hypoxic tumor area (135). Moreover, this is not only a migratory advantage. Functional assays show that Vδ1^+^ T cells that are tissue-resident and derived from the non-small cell lung cancer (NSCLC) microenvironment maintain normal effector functions under hypoxia (118).

In summary, Vδ1^+^ T cells are inherently hypoxic-adapted and can maintain their key therapeutic functions, including survival, infiltration, and effector functions within the adverse TME. Thus, this trait makes Vδ1^+^ T cells superior immunotherapies for targeting hypoxic solid tumors. Consequently, understanding the specific mechanisms of this adaptation is essential not only to understand their functional benefit, but it also represents an important research frontier in the quest to optimize next-generation TME-resistant cellular immunotherapies.

## 4 Vδ1^+^ T cell-based tumor immunotherapies

**Figure 2:**
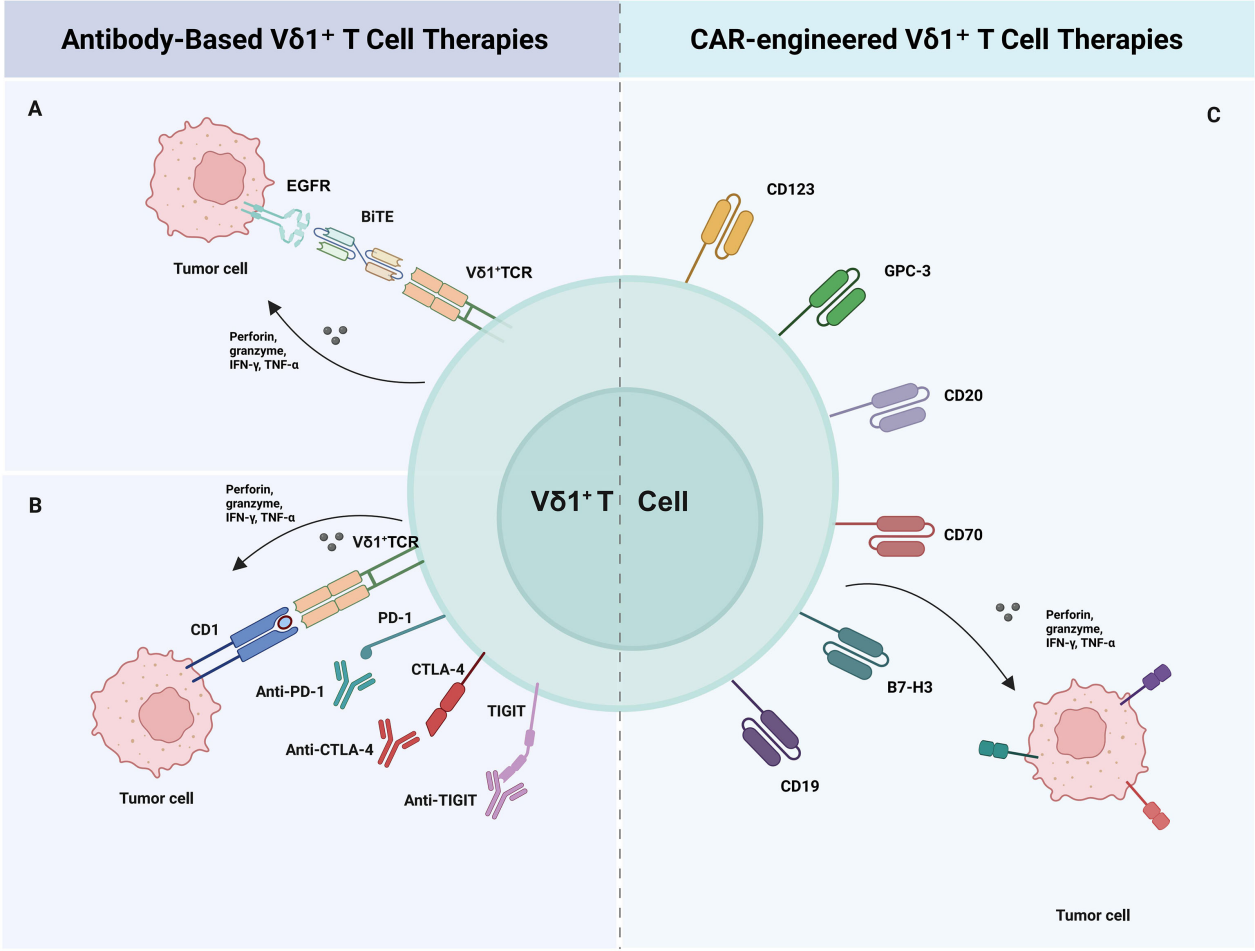
Targeted therapeutic strategies for Vδ1^+^ T cell immunotherapy. **(A)** Antibody-based therapies: Bispecific T cell engagers (BiTEs) bridge Vδ1^+^ TCR to tumor cell antigens (*e.g.*, EGFR), triggering release of cytotoxic granules (perforin, granzyme) and cytokines (IFN-γ, TNF-α). **(B)** Checkpoint blockade therapies: Anti-PD-1, anti-CTLA-4, and anti-TIGIT antibodies block inhibitory signaling, restoring Vδ1^+^ T cell cytotoxicity. TCR-mediated recognition of CD1-presented antigens is also shown. **(C)** CAR-engineered Vδ1^+^ T cell therapies, Genetically modified Vδ1^+^ T cells expressing chimeric antigen receptors (CARs) targeting antigens including CD123, GPC-3, CD20, CD70, B7-H3, and CD19 mediate tumor cell killing. BiTE, bispecific T cell engager; CAR, chimeric antigen receptor; TCR, T cell receptor; PD-1, programmed cell death protein 1; CTLA-4, cytotoxic T-lymphocyte-associated protein 4; TIGIT, T cell immunoreceptor with Ig and ITIM domains; EGFR, epidermal growth factor receptor.

Vδ1^+^T cells exhibit spontaneous anti-tumor activity in cancer immunotherapy. The *in vitro* cytotoxicity and the anti-leukemic effects of these cells were reported many years ago during bone marrow transplantation (136–139). The relatively limited clinical translation of these cells, however, was constrained for a long time due to the lack of efficient isolation and large-scale expansion technologies. Recent advances in amplification and gene-editing techniques have facilitated Vδ1^+^ T cell-based immunotherapy towards early clinical exploration, leading to the emergence of a range of strategies such as antibody-based therapies and therapies based on the adoptive cell transfer (ACT) (**Table 2**; **Figure 2**).

**Table 2:** Preclinical and clinical studies based on Vδ1^+^ T cells.

Company/institution	Product name	Therapeutic strategy	Target	Clinical trial & registration number	Indication	Reference
PureTech Health	LYT-210	Fully humanized monoclonal antibody	Vδ1^+^ TCR	Preclinical	Cancer (targeting pathogenic/immunosuppressive Vδ1^+^ γδ T cell subsets)	(144)
Takeda	ADT3	Bispecific T cell engager	Vδ1^+^ TCR × EGFR	Preclinical	EGFR-positive solid tumors	(148)
GammaDelta Therapeutics (acquired by Takeda)	GDX012	Unmodified adoptive Vδ1^+^ T cell therapy	None	Phase I (NCT05001451)	Acute myeloid leukemia with minimal residual disease	(154–157)
University of Toronto	Pembrolizumab (anti-PD-1)	Checkpoint inhibitor therapy	PD-1	Preclinical	Merkel cell carcinoma	(119)
Leiden University Medical Center	Anti-PD-1 + Anti-CTLA-4	Checkpoint inhibitor therapy	PD-1, CTLA-4	Preclinical	β2m-deficient, MMR-d colorectal cancer	(14)
Medical University of Vienna	Anti-TIGIT mAb	Checkpoint inhibitor therapy	TIGIT	Preclinical	MSS colorectal cancer	(112)
Takeda	TK012	Unmodified adoptive Vδ1^+^ T cell therapy	None	Phase 1/2a (NCT05886491)	Relapsed/refractory acute myeloid leukemia	(7, 158)
University of Barcelona	NA	CAR-modified Vδ1^+^ T cell therapy	CD123	Preclinical	Acute myeloid leukemia	(155)
Adicet Bio	ADI-002	CAR-modified Vδ1^+^ T cell therapy	GPC-3	Preclinical	GPC-3-positive hepatocellular carcinoma	(159, 160)
	ADI-001	CAR-modified Vδ1^+^ T cell therapy	CD20	Phase I (NCT04735471, NCT04911478)	B-cell malignancies	(161–164)
	ADI-270	CAR-modified Vδ1^+^ T cell therapy	CD70	Phase 1/2(NCT06480565)	R/R ccRCC	(165–167)
PersonGen Bio	UTAA06 Injection	CAR-modified Vδ1^+^ T cell therapy	B7-H3	IIT (NCT06372236)	B7-H3-positive relapsed or advanced malignant solid tumors	(168)
	UTAA09 Injection	CAR-modified Vδ1^+^ T cell therapy	CD19	IIT (NCT06503211)	R/R B-NHL	(169)
				Phase I	R/R B-ALL	(170)

This table summarizes representative preclinical and clinical studies of Vδ1^+^ T cell-based therapies, including monoclonal antibodies, bispecific T cell engagers, checkpoint inhibitor combinations, adoptive transfer, and CAR-modified strategies.

BiTE, bispecific T cell engager; CAR, chimeric antigen receptor; CPI, checkpoint inhibitor; EGFR, epidermal growth factor receptor; GPC-3, Glypican-3; IIT, investigator-initiated trial; mAb, monoclonal antibody; MMR-d, mismatch repair-deficient; MSS, microsatellite-stable; PD-1, programmed cell death protein 1; CTLA-4, cytotoxic T-lymphocyte-associated protein 4; TIGIT, T cell immunoreceptor with Ig and ITIM domains; TCR, T cell receptor; Vδ1^+^ T cells, V delta 1-positive T cells; ccRCC, clear cell renal cell carcinoma; R/R, relapsed/refractory; NHL, non-Hodgkin lymphoma; ALL, acute lymphoblastic leukemia.

### 4.1 Antibody-based therapies

#### 4.1.1 Monoclonal antibodies

IL-17 secretion from certain Vδ1^+^ T cell subsets may contribute to tumor immune evasion by fostering an immunosuppressive tumor microenvironment. Specifically, IL-17 promotes the recruitment of myeloid-derived suppressor cells (MDSCs) and M2-polarized macrophages, whose accumulation suppresses cytotoxic T cell infiltration and effector function, thereby facilitating immune escape.IL-17 is known to correlate with poor prognosis in various cancers (140–143). PureTech Health’s LYT-210 is a fully humanized monoclonal antibody against the Vδ1^+^ TCR. This therapeutic candidate aims to selectively deplete immunosuppressive Vδ1^+^ T cell populations (144). This strategy has been proposed as a novel approach to modulating pathogenic/pro-tumorigenic subsets by targeting immunosuppressive Vδ1^+^ T cell subsets (144).

#### 4.1.2 Bispecific T cell engagers

Bispecific T cell engagers (BiTEs) are designed to redirect T cells toward tumor cells through simultaneous binding to a tumor-associated antigen and a T cell receptor complex, thereby enabling proximity-dependent activation and cytotoxic function. This approach has garnered significant interest as a therapeutic strategy in the current landscape of immuno-oncology research (145–147).

ADT3 is a bispecific T cell engager developed by Takeda that redirects Vδ1^+^ T cells toward epidermal growth factor receptor (EGFR)-expressing tumor cells. Its activity is mediated by concurrent engagement of the Vδ1^+^ TCR and EGFR, promoting the formation of a functional immunological synapse between effector and target cells (148). Structurally, ADT3 comprises a Vδ1^+^ TCR-binding Fab domain and an engineered Fc region with bivalent EGFR-binding capacity (148). This bivalency enhances avidity and drives receptor clustering, thereby stabilizing cell–cell interactions and supporting the formation of a productive immune synapse (149). Stable synapse formation underpins sustained downstream signaling and efficient effector function, including cytotoxicity and cytokine production, whereas insufficient synapse stability is associated with impaired target cell killing (150, 151). Preclinical studies demonstrate that ADT3 induces expansion and activation of Vδ1^+^ T cells and mediates potent cytotoxicity against EGFR-positive tumor cells (148). Collectively, these findings highlight the potential of bispecific strategies to harness tissue-resident Vδ1^+^ T cells for targeted antitumor responses.

#### 4.1.3 Checkpoint Inhibitors

##### 4.1.3.1 Restoration of tumor-Infiltrating Vδ1^+^ T cells by PD-1 inhibitors

The expression of PD-1 on Vδ1^+^ T cells enable their functional restoration *via* checkpoint inhibitors, providing a unique clinical opportunity to enhance anti-tumor immunity. At the clinical level, Lien et al. reported a Merkel cell carcinoma patient who achieved a complete response to pembrolizumab, a PD-1 monoclonal antibody, with functional Vδ1^+^ T cells identified as critical mediators of the therapeutic effect (119). Furthermore, dual blockade of PD-1 and cytotoxic T-lymphocyte-associated protein 4 (CTLA-4) has been shown to promote the expansion of Vδ1^+^ T cells in patients with β2-microglobulin-deficient, MMR-d colorectal cancer, highlighting the enhanced therapeutic potential of γδ T cells through combined checkpoint inhibition (14). Collectively, these findings confirm that PD-1 blockade can effectively mobilize and reactivate Vδ1^+^ T cells, thereby contributing to clinical anti-tumor responses.

##### 4.1.3.2 Restoration of Vδ1^+^ T cell function by TIGIT inhibitors

Beyond PD-1, the TIGIT serves as another critical immunosuppressive checkpoint for Vδ1^+^ T cells. Stary et al. demonstrated that blocking TIGIT-NECTIN2 interaction with an anti-TIGIT monoclonal antibody significantly restored the cytolytic activity of dysfunctional Vδ1^+^ T cells in MSS CRC patients (120). This finding provides direct preclinical proof-of-concept for combining anti-TIGIT antibodies with Vδ1^+^ T cell-based therapies, particularly in MSS CRC, a tumor type that remains largely refractory to conventional immunotherapies.

### 4.2 Adoptive cell therapy

#### 4.2.1 *In vitro* expansion technology of Vδ1^+^ T cells

Repeated research studies by several groups have succeeded in developing effective systems for the *in vitro* expansion of Vδ1^+^ T cells (34, 57, 58, 79, 152, 153). However, Almeida et al.’s Delta One T (DOT) cell technology (59) represents one of the more advanced and extensively characterized expansion strategies. Under optimized differentiation and amplification conditions, this protocol was reported to achieve substantial expansion of Vδ1^+^ T cells within approximately three weeks. Notably, the resulting DOT cell product exhibits an immunophenotype characterized by increased expression of activating natural killer receptors, including NKp30 and NKG2D, together with relatively low expression of inhibitory receptors such as PD-1 and CTLA-4, which may collectively contribute to reduced inhibitory signaling, decreased T cell exhaustion, and enhanced cytotoxic function. Consistent with these features, DOT cell–based approaches have demonstrated anti-leukemic activity in multiple acute myeloid leukemia patient-derived xenograft models (154, 155).

#### 4.2.2 Unmodified ACT

Using its DOT cell expansion platform, GammaDelta Therapeutics—subsequently acquired by Takeda—developed GDX012, a non-genetically modified adoptive cell therapy. A first-in-human Phase I clinical trial (NCT05001451) was initiated in patients with acute myeloid leukemia (AML) with minimal residual disease. In this study, patients received lymphodepleting conditioning with fludarabine and cyclophosphamide followed by GDX012 infusion. The trial was designed to evaluate safety and tolerability, determine the maximum tolerated dose, and assess preliminary anti-leukemic activity (156). Subsequently, a second clinical study (NCT05886491) was initiated in patients with relapsed or refractory AML. According to publicly available registry records, both trials have since been terminated. No safety-related concerns were reported in the available data, and the reasons for discontinuation were not explicitly disclosed (7, 157, 158).

#### 4.2.3 Chimeric antigen receptor (CAR)-modified ACT

Owing to their MHC-independent recognition and functional properties, as well as their reported cytotoxic activity in preclinical settings, Vδ1^+^ T cells have attracted interest as a platform for CAR engineering. This strategy has developed rapidly, with a growing number of investigational CAR therapies targeting diverse antigens in both hematologic and solid tumors, several of which are making good progress in preclinical and clinical studies.

##### 4.2.3.1 CD123-targeted CAR Vδ1^+^ T cells

Recent studies have reported that allogeneic CAR Vδ1^+^ T cells (CAR DOT cells) utilize their inherent properties of recognition without MHC restriction to exhibit measurable activity. The cytotoxicity of CD123-specific CAR-transduced Vδ1^+^ T cells against AML cell lines and primary patient samples *in vitro* is significantly higher than that of unmodified Vδ1^+^ T cell controls. According to *in vivo* studies, a single injection of CAR DOT cells with IL-15 was associated with delayed AML progression in mouse models. These cells show sustained anti-leukemic activity upon tumor rechallenge, demonstrating functional persistence (155).

##### 4.2.3.2 ADI-002: GPC-3-positive liver cancer-targeted CAR Vδ1^+^ T cells

Adicet Bio’s ADI-002 is a CAR Vδ1^+^ T cell treatment that targets Glypican-3 (GPC-3)-positive liver cancer. It is currently at the investigational new drug (IND) application stage (159). This genetically engineered therapy allows Vδ1^+^ T cells to co-express a GPC-3-specific CAR and soluble IL-15, intended to support their expansion and persistence. The modified cells are able to expand *in vitro* and exhibit cytotoxic activity against hepatocellular carcinoma cell lines. The co-expression of IL-15 supports the proliferation of T cells and the maintenance of cytotoxicity and was associated with detectable *in vivo* anti-tumor responses without evidence of GVHD in preclinical models (160).

##### 4.2.3.3 ADI-001: CD20-targeted CAR Vδ1^+^ T cells

Adicet Bio’s ADI-001 is an allogeneic cell therapy with CAR Vδ1^+^ T cells that target CD20. It has been reported to exhibit cytotoxic activity in preclinical models, and Phase I trials have been initiated (161, 162). This candidate is designed as a fully humanized anti-CD20 monoclonal antibody which also co-expresses multiple NKRs and chemokine receptors. The design enables the activation of innate and adaptive mechanisms for anti-tumor immune responses (162).

According to preclinical studies, ADI-001 is capable of inducing tumor cell lysis and stimulating pro-inflammatory cytokine production *in vitro*. Moreover, it has been shown to enhance *in vivo* tumor growth inhibition without GVHD, without evidence of GVHD in preclinical studies (162).

Phase I trials (NCT04735471, NCT04911478) in relapsed or refractory B-cell non-Hodgkin lymphoma (R/R B-NHL) indicate that ADI-001 was generally well tolerated without dose-limiting toxicities or GVHD (163, 164). The efficacy results are encouraging, with objective responses reported in early interim analyses. Significantly, the ORR and CR rates were reported in a limited number of patients previously treated with anti-CD19 CAR-T, indicating the potential to overcome CD19 resistance (161). The clinical efficacy of ADI-001 requires further follow-up, although its therapeutic potential in B-cell malignancies, including CD19-resistant cases, is supported by these findings.

##### 4.2.3.4 ADI-270: CD70-positive malignancy-targeted CAR Vδ1^+^ T cells

Adicet Bio has developed ADI-270, an allogeneic CAR Vδ1^+^ T cell therapy for the treatment of CD70-positive malignancies. To enhance efficacy, its design integrates several strategies including a third-generation CAR, CD70 recognition *via* CD27, and a dominant-negative TGF-β receptor II (dnTGFβRII) to resist immunosuppression (165). A Phase I/II clinical trial (NCT06480565) is underway to evaluate the safety, tolerability, and preliminary anti-tumor activity of ADI-270 in patients with relapsed or refractory clear cell renal cell carcinoma (ccRCC) (166, 167).

##### 4.2.3.5 UTAA06 Injection: B7-H3-targeted UCAR Vδ1^+^ T cells

In the field of domestic cell therapy, PersonGen Bio has developed UTAA06 Injection, a universal off-the-shelf B7-H3 CAR Vδ1^+^ T cell product. The target population is patients with advanced malignant solid tumors, specifically those with tumor cell membrane B7-H3 expression intensity of 1+ or more and a positive staining proportion of ≥50%. Recently, an investigator-initiated trial from the Peking University in China reported the first investigator-initiated trial (IIT) (NCT06372236) that gave its first infusion for an ovarian cancer (OC) patient. According to early data, there have been no dose-limiting toxicities reported at the time of initial treatment, suggesting some early support for the candidate’s safety (168).

##### 4.2.3.6 UTAA09 injection: CD19-targeted UCAR Vδ1^+^ T cells

UTAA09 is also a universal off-the-shelf CAR-T cell injection based on Vδ1+ T cells and targeting CD19, developed by PersonGen Bio. In 2024, the First Affiliated Hospital of Nanjing Medical University began a IIT trial (NCT06503211) to explore its preliminary safety and efficacy in patients with R/R B-NHL (169). In April 2025, the approval of UTAA09 Injection by China’s National Medical Products Administration (NMPA) was granted to allow progression into clinical trials for adult patients with relapsed or refractory B-cell acute lymphoblastic leukemia (R/R B-ALL) (170).

## 5 Challenges and future directions of Vδ1^+^ T cell immunotherapy

Vδ1^+^ T cells represent a promising platform for tumor immunotherapy, characterized by MHC-unrestricted and multi-modal antigen recognition. Their intrinsic advantages, including broad targetability, tissue residency, efficient tumor infiltration, and functional persistence within the tumor microenvironment, provide a strong foundation for therapeutic development. These features have driven the emergence of antibody-based strategies and engineered cellular therapies, particularly CAR-based approaches, and highlight their growing translational potential.

Vδ1^+^ T cells, in addition to sharing NKG2D expression with Vγ9Vδ2 T cells (55–58, 68, 69), exhibit a broader receptor repertoire that includes natural cytotoxicity receptors such as NKp30 and NKp46 (29, 59). Their TCRs are capable of recognizing CD1 molecules (50–54) as well as a range of non-classical ligands, including MR1 (60) and EphA2 (61, 62), thereby conferring a flexibility in target recognition (**Figure 1**; **Table 1**). Notably, these distinct recognition pathways differ not only in ligand specificity, but also in signaling context, activation thresholds, and downstream transcriptional outputs. Accumulating evidence indicates that signaling derived from the TCR and NKG2D can act in concert in γδT cells (171). Their co-engagement has been shown to enhance intracellular calcium mobilization (172) and may facilitate downstream transcriptional activation, ultimately amplifying cytokine production and accelerating degranulation kinetics. However, despite these observations, the mechanisms underlying signal integration across these pathways remain incompletely understood, particularly with respect to how they influence the balance between effector and regulatory programs. This limitation may constrain the rational optimization of Vδ1^+^ T cell–based therapies, as receptor crosstalk is likely to critically shape activation thresholds, functional persistence, and susceptibility to exhaustion. Further elucidation of these signaling networks will therefore be essential for optimizing therapeutic strategies and improving the precision of Vδ1^+^ T cell–based interventions.

Across multiple tumor types, Vδ1^+^ T cells exhibit a exhaustion-like phenotype, accompanied by the upregulation of inhibitory receptors such as PD-1 (14, 119), TIGIT (120), and CTLA-4 (14). In melanoma, PD-1^+^Vδ1^+^ T cells retain responsiveness to innate and TCR-mediated stimulation and can be partially reinvigorated by PD-1/PD-L1 blockade (119). Dual PD-1 and CTLA-4 blockade expands Vδ1^+^ T cells in β2-microglobulin−deficient, MMR−d colorectal cancer, supporting the enhanced efficacy of combined checkpoint inhibition in γδ T cell−mediated immunity (14) (**Figure 2**; **Table 2**). From a translational perspective, co-targeting may offer advantages over single-agent checkpoint blockade. In parallel, shRNA-mediated checkpoint knockdown may provide more sustained inhibition (173), with the added advantage that knockdown efficiency can be tuned to relieve inhibitory signaling without inducing excessive activation. Bispecific engagers can confer tumor specificity on Vδ1^+^ T cells. For example, ADT3 links Vδ1^+^ T cells to tumor antigens such as EGFR, promoting activation, expansion, and cytotoxicity (148) (**Figure 2**; **Table 2**). Future strategies may incorporate dual-antigen targeting and checkpoint-blocking modules to enhance efficacy, particularly in solid tumors with antigen heterogeneity and immunosuppressive microenvironments.

Although unmodified Vδ1^+^ T cells show therapeutic potential, their efficacy remains limited by antigen escape and functional impairment within the tumor microenvironment. CAR engineering provides a powerful approach to enhance antigen specificity (174, 175), and CAR-Vδ1^+^ T cells targeting diverse antigens are currently being evaluated in clinical studies across both hematologic malignancies and solid tumors (**Figure 2**; **Table 2**). However, CAR-driven activation is typically strong and may accelerate exhaustion under conditions of chronic antigen stimulation (176). To address this, multiple strategies have been explored in conventional CAR-T cells, including optimization of co-stimulatory domains (177), modulation of transcriptional programs (178, 179), and enhancement of metabolic fitness (180, 181). These strategies hold promise for extension to Vδ1^+^ T cells to further enhance their antitumor function. Previous studies have reported that, compared with conventional αβ T cells, γδ T cells, including the Vδ1^+^ subset, generally exhibit lower transduction efficiency with lentiviral vectors (182). Despite ongoing efforts to optimize transduction conditions, such as improving activation protocols, modulating cell cycle status, and applying transduction enhancers, overall efficiency in γδ T cells remains suboptimal (183). To address this limitation, engineering of the viral envelope to enhance targeting represents a promising strategy (184). For example, incorporation of targeting modules, such as scFv fragments, into envelope proteins can improve virus–cell interactions and thereby increase transduction efficiency. Furthermore, the addition of targeting modules not only improves transduction efficiency in Vδ1^+^ T cells but also lays the foundation for the development of *in vivo* CAR-Vδ1 T cell therapies.

In summary, this review highlights the unique biological features and therapeutic potential of Vδ1^+^ T cells in cancer immunotherapy. Their MHC-unrestricted antigen recognition, broad receptor repertoire, and intrinsic adaptability within the tumor microenvironment collectively position them as a promising platform for next-generation cellular therapies. In this context, we further emphasize several key aspects that warrant deeper investigation, including the mechanisms of receptor-mediated signal integration, the functional implications of immune checkpoint expression, the optimization of genetic engineering strategies, and the improvement of transduction efficiency during cell manufacturing. Addressing these areas will be critical for advancing our mechanistic understanding of Vδ1^+^ T cells and facilitating their clinical translation.

## 6 Methods

This systematic review was performed in accordance with the PRISMA 2020 guidelines. A comprehensive literature search was conducted across multiple databases, including PubMed, Web of Science Core Collection, Scopus, Annual Reviews, and Google Scholar. The literature retrieval cutoff date was April 2026.The Boolean search strategy was defined as follows: (“Vδ1 T cells” OR “γδ T cells” OR “gamma delta T cells”) AND (“tumor” OR “cancer” OR “tumor immunity”) AND (“immunotherapy” OR “adoptive cell therapy” OR “CAR-T” OR “NKG2D” OR “butyrophilin” OR “EPCR” OR “EphA2” OR “tumor microenvironment” OR “immune evasion” OR “clinical trials”). A total of 1213 records were initially identified from the above databases. Supplementary searches were performed on clinical trial registries (ClinicalTrials.gov, the Center for Drug Evaluation [CDE] database) and biopharmaceutical company websites, yielding 8 additional records. After removing 170 duplicates, 1051 unique records were screened by title and abstract. Of these, 709 records were excluded as irrelevant, and 342 full-text articles were retrieved for eligibility assessment. Full-text articles were excluded if they (1): primarily focused on Vγ9Vδ2 T cells or other immune cell subsets rather than Vδ1^+^ T cells (n = 120) (2); investigated Vδ1^+^ T cells in non-tumor contexts (n = 38). In total, 158 records were excluded during full-text evaluation.

**Figure 3:**
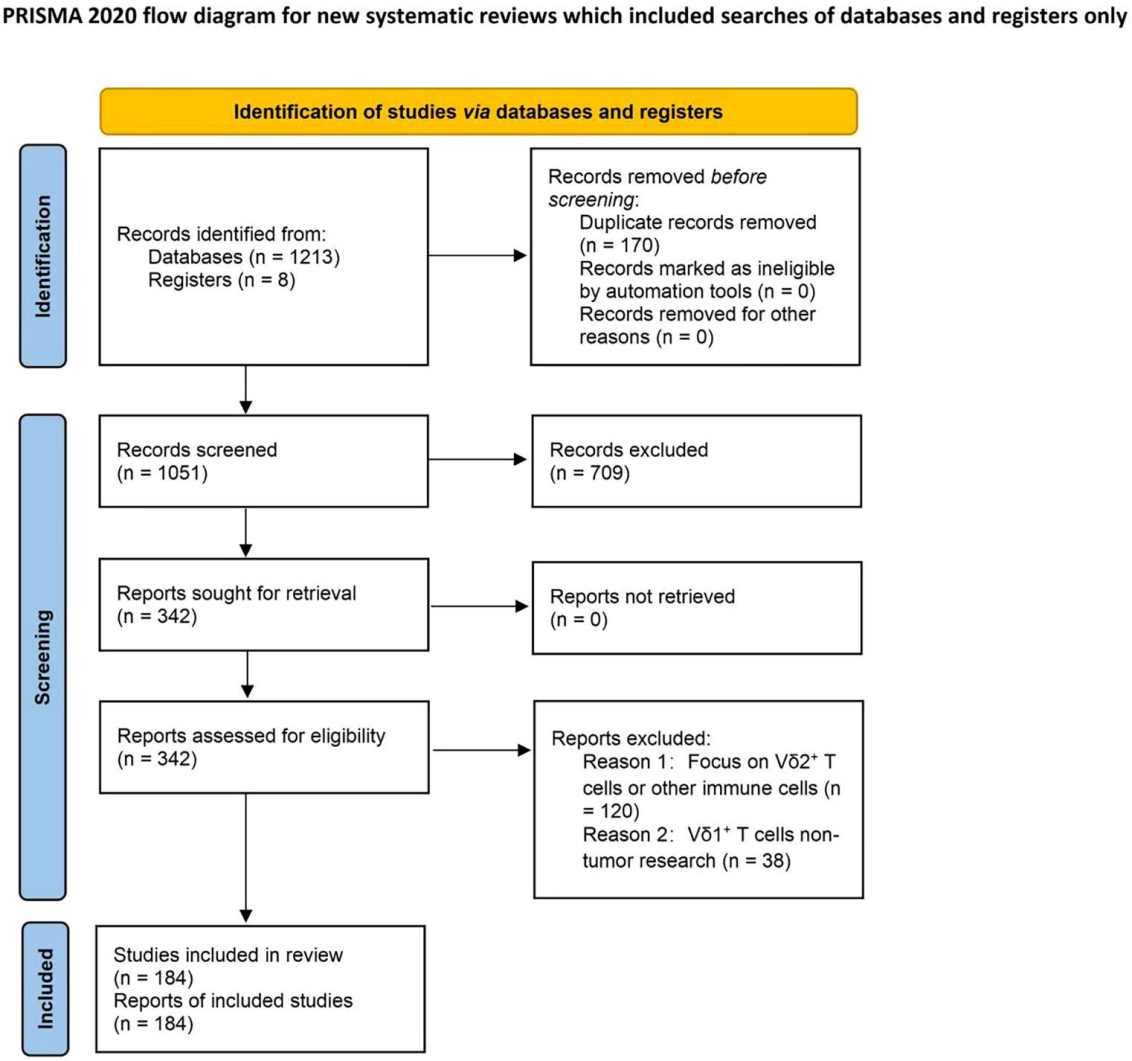
PRISMA 2020 flow diagram showing the literature screening procedure for studies on Vδ1^+^ T cell-based cancer immunotherapy.

All literature screening and selection processes were completed independently. Any inconsistencies in study inclusion were settled by collective discussion. Given the nature of this narrative review, a formal risk-of-bias assessment was not conducted. Finally, 184 eligible articles were included in this review. The PRISMA flow diagram is shown in **Figure 3**.
